# Increased Peripheral Venous Catheter Bloodstream Infections during COVID-19 Pandemic, Switzerland

**DOI:** 10.3201/eid3001.230183

**Published:** 2024-01

**Authors:** Marie-Céline Zanella, Eva Pianca, Gaud Catho, Basilice Obama, Marlieke E.A. De Kraker, Aude Nguyen, Marie-Noëlle Chraiti, Jonathan Sobel, Loïc Fortchantre, Stephan Harbarth, Mohamed Abbas, Niccolò Buetti

**Affiliations:** Infection Control Programme and World Health Organization Collaborating Center, Geneva University Hospitals and Faculty of Medicine, Geneva, Switzerland (M.-C. Zanella, E. Pianca, G. Catho, B. Obama, M.E.A. De Kraker, A. Nguyen, M.-N. Chraiti, L. Fortchantre, S. Harbarth, M. Abbas, N. Buetti);; University of Geneva, Geneva (J. Sobel);; MRC Centre for Global Infectious Disease Analysis, Jameel Institute, School of Public Health, Imperial College London, London, UK (M. Abbas);; Université Paris-Cité, Paris, France (N. Buetti)

**Keywords:** COVID-19, respiratory infections, severe acute respiratory syndrome coronavirus 2, SARS-CoV-2, SARS, coronavirus disease, zoonoses, viruses, coronavirus, catheter, peripheral-line, bloodstream infection, surveillance, Switzerland, bacteria

## Abstract

Studies suggest that central venous catheter bloodstream infections (BSIs) increased during the COVID-19 pandemic. We investigated catheter-related BSIs in Switzerland and found peripheral venous catheter (PVC) BSI incidence increased during 2021–2022 compared with 2020. These findings should raise awareness of PVC-associated BSIs and prompt inclusion of PVC BSIs in surveillance systems.

Peripheral intravenous catheters (PVCs) and central venous catheters (CVCs) are frequently used in hospitalized patients. Estimates from global device sales illustrated that ≈1.2 billion PVCs are used worldwide annually (*1*,*2*). PVC-related complications include phlebitis, hematoma, and extravasation (*3*,*4*). PVC-associated bloodstream infections (BSIs) often are disregarded in surveillance systems because of low incidence (*5*,*6*). However, because PVCs are widely used in hospitalized patients, the burden of PVC-associated or related BSIs might still be substantial. In contrast, only 10% of acute care inpatients have a CVC inserted (*7*), but the incidence of BSIs associated with CVCs is higher than that for PVCs, likely because infection prevention strategies mostly focus on CVCs.

Several studies have shown that intravascular catheter infections increased during the COVID-19 pandemic (*8*–*11*). Those studies mainly focused on BSIs associated with CVCs. COVID-19 might have substantially affected the frequency of PVC infections, but published reports are lacking. To assess the incidence of BSIs associated with or related to intravenous catheters, we used a large prospective database to study BSIs by catheter type during the COVID-19 pandemic in Switzerland.

## The Study

We performed a cohort study at Geneva University Hospitals (HUG), a large network of tertiary care centers in Switzerland. HUG includes 5 rehabilitation or palliative care sites and 1 acute care, 1 geriatric, 1 pediatric, 1 gynecology-obstetrics, and 1 psychiatric site. HUG has ≈2,100 beds and receives 60,000 hospital admissions per year.

We included all patients hospitalized during January 1, 2020–December 31, 2022. All hospital-acquired BSIs during that timeframe were investigated as part of prospective hospital-wide surveillance, which has been conducted for >25 years by the HUG infection control program. We limited the analysis to catheter-related or -associated BSIs (CRABSIs), comprising catheter-related BSI (CRBSI) and catheter-associated BSI (CABSI). We classified CRABSI that were attributed to PVC, short-term CVC, and long-term CVC. The infection control program routinely collects patient data from CRABSI, including onset date, age, sex, ward of acquisition, catheter type, and microorganism identified.

The primary outcomes (i.e., CRABSI) were based on European Centre for Disease Prevention and Control definitions (*12*). A CRBSI required a positive blood culture <48 hours after catheter removal and the same microorganism isolated from a quantitative catheter tip culture or the same microorganism isolated in a culture from pus collected from a catheter site (Appendix). A CABSI required a positive blood culture occurring from time of insertion until 48 hours after catheter removal, resolution of symptoms within 48 hours after catheter removal, and no other infectious focus. We also tracked details of COVID-19 infections reported in the hospital system (Appendix).

**Figure 1:**
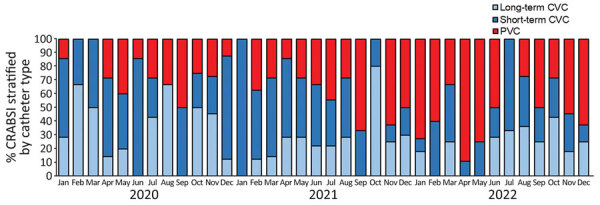
Percentage of intravascular catheter infections stratified by catheter type in study of intravascular catheter bloodstream infections during the COVID-19 pandemic, Switzerland, January 1, 2020–December 31, 2022. CRABSI, catheter-related or -associated bloodstream infections; CVC, central venous catheter; PVC, peripheral venous catheter.

We used patient-days as the main denominator, which we extracted from the electronic record system. We used a 5-step statistical plan. First, we determined the total monthly incidence of CRABSI, and CRABSI attributed to PVC, short-term CVC, and long-term CVC per 1,000 patient-days (Figure 1). Second, we evaluated incidence rate ratios (IRRs) for intravascular catheter infections stratified for catheter type for 2021 and 2022 by segmented Poisson regression models using aggregated monthly data and used 2020 as the referent and patient-days as the offset. We tested overdispersion by using the likelihood ratio test and subsequently fit a negative binomial model, if required. Third, we compared patient and microbiologic characteristics of CRABSI attributed to PVC between the different periods using χ^2^ test for categorical variables and Kruskal-Wallis test for continuous variables. Fourth, we determined the number of PVCs and PVCs in situ >4 days inserted per month. Fifth, we performed a sensitivity analysis by using catheter-days as a denominator for CRABSI attributed to PVC and CVC.

We used SAS version 9.4 (SAS Institute, Inc., https://www.sas.com) to perform all analyses and considered p<0.05 statistically significant. This analysis complies with STROBE guidelines for observational studies (*13*).

During the study period, a total of 179,463 patients were hospitalized at HUG, corresponding to 1,978,177 patient-days. We included 249 CRABSI episodes. We observed 90 CRABSI attributed to PVC, 94 attributed to short-term CVC, 74 attributed to long-term CVC, and 9 cases were possibly attributable to >1 intravascular catheter. Overall, the median age of patients with a CRABSI was 61 (interquartile range [IQR] 47–73) years; 62.3% (n = 155) were male and 37.7% (n = 94) were female. Most (37.8%, n = 94) CRABSI were caused by coagulase-negative staphylococci (Appendix Table 1).

CRABSI incidence remained stable during the study period, but we observed peaks in CRABSI attributed to short-term and long-term CVC during November 2021–January 2022 (Appendix Figure 1). Of note, incidence of CRABSI attributed to PVC increased during late 2021 and in 2022. Similarly, the proportion of CRABSI attributed to PVC among all intravascular catheter infections increased during late 2021 and in 2022 (Figure 1).

**Figure 2:**
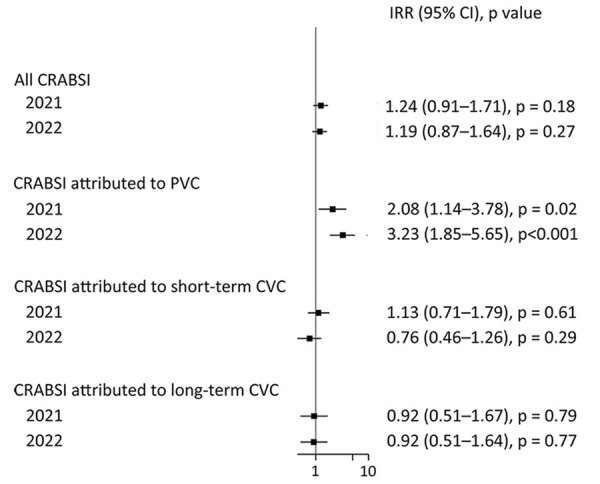
Incidence rate ratios per 1,000 patient days in a study of intravascular catheter bloodstream infections during the COVID-19 pandemic, Switzerland, January 1, 2020–December 31, 2022. Squares indicate IRRs, bars indicate 95% CIs. Patient-days were used as the denominator; 2020 rates were used as the referent. BSI, bloodstream infection; CRABSI, catheter related or associated bloodstream infections; CVC, central venous catheter; IRR, incidence rate ratio; PVC, peripheral venous catheter.

Overall, compared with 2020, IRRs for CRABSI did not significantly increase in 2021 (IRR 1.24, 95% CI 0.91–1.71; p = 0.18) and 2022 (IRR 1.19, 95% CI 0.87–1.64; p = 0.27) (Figure 2; Appendix Table 2). By contrast, rates of CRABSI attributed to PVC significantly increased during 2021 (IRR 2.08, 95% CI 1.14–3.78; p = 0.02) and 2022 (IRR 3.23, 95% CI 1.85–5.65; p<0.01) compared with 2020. Rates of CRABSI attributed to short-term and long-term CVC did not show statistically significant changes (Figure 2; Appendix Table 2).

Among patients with CRABSI attributed to PVC, we did not observe statistically significant differences for sex, age, ward of acquisition, or microorganism distribution (Appendix Table 1). We observed similar results for short-term and long-term CVC (Appendix Table 1). Furthermore, the monthly number of CVCs and PVCs inserted, and PVCs in situ >96 hours did not change over time (Appendix Table 3, Figures 2, 3). A sensitivity analysis using catheter-days as a denominator yielded similar results (Appendix Figure 4).

## Conclusions

This study showed that CRABSI attributed to PVC increased during the 2021–2022 compared with 2020. Studies in different countries showed that CVC-related BSIs increased during the COVID-19 pandemic (*10*,*11*), but no data on PVC-related infections are available. 

Several hypotheses might explain these findings. First, ward of acquisition and microorganism distributions from 2020–2022 did not substantially change among PVC-related BSIs. Nevertheless, we observed a nonsignificant increase of PVC-attributed CRABSI due to coagulase-negative staphylococci in surgery wards in 2022. Moreover, we did not observe a significant increase of blood culture contaminations during 2021–2022 compared with 2020 (*14*). Second, according to our institutional recommendations, PVCs should be routinely changed every 4 days. We did not observe an increase of PVCs inserted for >96 h, suggesting adequate compliance to that preventive measure (Appendix). Recent unpublished data from France showed similar alarming results in the surveillance system of devices associated infections (*15*).

Our study’s first limitation is that the study was single-center, limiting the generalizability of the results; however, HUG comprises several different sites, thus increasing the diversity of the patient population. Moreover, our data cannot be generalized to centers that routinely use midline catheters or that routinely use other infection control strategies, such as chlorhexidine-gluconate bathing post-CVC insertion or use of impregnated dressings. Second, we did not include confounders such as site of insertion, emergent versus elective insertions, immunocompromised states, chronic illnesses, body mass index, and nurse-to-patient ratio in our analysis. Third, our primary outcome, CRABSI, did not include pulmonary arterial, peripheral arterial, and umbilical arterial catheter infections.

In conclusion, our findings show that CRABSI attributed to PVC significantly increased during 2021–2022 in HUG. The observed increasing incidence of CRABSI attributed to PVC should raise awareness and warrants inclusion of PVC-related BSIs in national surveillance systems.

Appendix Additional information on increased peripheral intravascular catheter bloodstream infections during the COVID-19 pandemic, Switzerland.
