# Supplementary material for: Increased Peripheral Venous Catheter Bloodstream Infections during COVID-19 Pandemic, Switzerland
Source: Emerg Infect Dis. 2024 Jan;30(1):159–62. doi: 10.3201/eid3001.230183 (PMC10756358; doi:10.3201/eid3001.230183)
Supplement: Appendix — Additional information on increased peripheral intravascular catheter bloodstream infections during the COVID-19 pandemic, Switzerland. [file 23-0183-Techapp-s1.pdf]

Article DOI: https://doi.org/10.3201/eid3001.230183

EID cannot ensure accessibility for supplementary materials supplied by authors. Readers who have difficulty accessing supplementary content should contact the authors for assistance.

Increased Peripheral Venous Catheter Bloodstream Infections during COVID-19 Pandemic, Switzerland

Appendix

Included Intravascular Catheters

Intravascular catheters included in this study were peripheral vascular catheters (PVCs), and short-term and long-term central venous catheters (CVCs). Long-term CVC were tunneled catheters, hemodialysis catheters, totally implanted ports and peripherally inserted central catheter (PICC) lines. No midline catheters are inserted at HUG.

Definitions

For more than 25 years, the University of Geneva Hospitals system has performed a hospital-wide prospective surveillance of all healthcare-associated blood stream infections (BSIs). For each hospital-associated BSI episode, data on source of infection, and clinical and microbiological characteristics are routinely collected by the infection prevention and control (IPC) team. In brief, each new positive blood culture result (even without definite microbiological results; i.e., prospectively) is routinely communicated from the central microbiology laboratory to IPC team members during working days (i.e., each potential healthcare-associated positive blood culture result is assigned to a member of the IPC team), who prospectively follow up and investigate sources of healthcare-associated episodes. Criteria for investigating episodes were: 1) occurrence more than 48 hours after hospital admission or previous hospitalization in the last 10 days, 2) surgery in the last 30 (nonimplant surgery) or 90 (implant surgery) days, 3) occurrence in neonatal units, 4) positive blood culture results in

patient obstetric wards, and 5) positive blood culture results in hemodialysis and onco-hematologic outpatients.

Classification of CVC- and PVC-related or -associated BSIs (i.e., CRABSI) were based on the European Centre for Disease Prevention and Control (ECDC) definitions (*1*). A catheter-related BSI (CRBSI) required a positive blood culture occurring from catheter insertion until 48h after catheter removal, with the same microorganism as isolated from a quantitative catheter tip culture $\geq 10^3$ CFU/ml (or semiquantitative catheter culture >15 colony forming unit) or a positive exit-site culture from pus. A catheter-associated BSI (CABSI) required a positive blood culture occurring from day of insertion until 48h after catheter removal, the resolution of symptoms in 48h after catheter removal and no other infectious focus. Common skin contaminants were included only if detected in at least two blood cultures within 48h and the patient had at least one sign or symptom of infection (chills, hypotension, or fever [>38.0°C]). Common skin contaminants were defined according to the National Healthcare Safety Network (NHSN) official list (e.g., *Corynebacterium* spp, *Bacillus* spp, *Micrococcus* spp, coagulase-negative staphylococci [CoNS], and *Propionibacterium* spp).

In comparison with the ECDC definitions that were applied in this study for the classification of CVC- and PVC-related or -associated BSIs (i.e., CRABSI), IDSA definitions consider only catheter tip cultures and differential time to positivity (DTP) for catheter-related infections (*2*), and US Centers for Disease Control and Prevention (CDC) CLABSI definitions do not consider catheter tip cultures and DTP (*3*,*4*).

COVID-19 Situation

In March 2020, HUG was transformed to accommodate the increase in COVID-19 patients (e.g., several units were transformed into COVID-19 dedicated units). The COVID-19 pandemic dramatically changed the patient population (non–COVID-19 were diverted to other "non–COVID-19" hospitals in the area) and influenced care procedures (e.g., healthcare workers [HCWs] were reallocated to different wards). Of note, infection prevention measures related to catheters remained similar during the different periods analyzed.

The COVID-19 hospitalizations were extracted using the Swiss hospital sentinel surveillance system (Hospital Based Surveillance CH-SUR), an ongoing surveillance system

since the beginning of the pandemic. The prospective surveillance of BSIs was continued even during the pandemic, from 1st January 2020 to 30th September 2022.

Infection Prevention Procedures

Institutional recommendations for PVC insertion and care were the following: (i) alcohol-containing 2% chlorhexidine-gluconate (CHG) was used for skin antisepsis at catheter insertion and during dressing changes; (ii) the selection of site of insertion was left to the discretion of the healthcare workers caring for the patient; (iii) semipermeable transparent dressings were used for all PVCs and changed when clinically indicated. PVCs were routinely replaced every 4 days. Institutional recommendations for CVC insertion and care were the following: (i) maximal sterile barrier precaution; (ii) alcohol-containing 2% CHG was used for skin antisepsis but no CHG-bathing was applied; (iii) the selection of site of insertion and the utilization of ultrasound guidance was left to the HCWs; (iv) semipermeable transparent dressings without antiseptic impregnation were used for all CVCs; (v) no impregnated catheters were routinely used. Soiled, leaking, or wet dressings were immediately changed. Infection prevention measures did not change during the study period.

Ethics

The hospital-acquired BSI surveillance is part of a mandatory indicator surveillance at HUG and thus, is considered as quality assurance. Only pseudonymized data has been processed, and therefore ethical board approval for data re-use is not required.

Additional Hypotheses for the Main Results of the Study

First, peaks of CRABSI attributed to PVC incidence were not concomitant with peaks of hospitalizations due to SARS-CoV-2 infections during 2020 and 2021, thus suggesting that hospital overcrowding may not have played a major role for the occurrence of CRABSI attributed to PVC. Second, we could hypothesize that the lack of compliance to other preventive measures or hospital reorganization, increased workload, staff shortage/turnover, as well as a reduced educational activities may have contributed to our observations in 2021–2022.

References

1. European Centre for Disease Prevention and Control. Surveillance of healthcare-associated infections and prevention indicators in European intensive care units [cited 2017 May 5]. https://www.ecdc.europa.eu/sites/default/files/documents/HAI-Net-ICU-protocol-v2.2_0.pdf

2. Mermel LA, Allon M, Bouza E, Craven DE, Flynn P, O'Grady NP, et al. Clinical practice guidelines for the diagnosis and management of intravascular catheter-related infection: 2009 Update by the Infectious Diseases Society of America. Clin Infect Dis. 2009;49:1–45. PubMed https://doi.org/10.1086/599376

3. US Centers for Disease Control and Prevention. Chapter 4: bloodstream infection (BSI) event. Central line-associated bloodstream infection (CLABSI) and non-central line-associated bloodstream infection [cited 2022 October 18]. https://www.cdc.gov/nhsn/psc/bsi/index.html

4. Buetti N, Timsit JF. Management and prevention of central venous catheter–related infections in the ICU. Semin Respir Crit Care Med. 2019;40:508–23. PubMed https://doi.org/10.1055/s-0039-1693705

Appendix Table 1. Characteristics of patients and microbiology of CRABSI attributed to PVC, short-term CVC, and long-term CVC during COVID-19 pandemic, Switzerland*

Characteristics	2020	2021	2022	p value
CRABSI attributed to PVC, n = 90†	N = 14	N = 31	N = 45	
Sex				
M	10 (71.4)	22 (71)	26 (57.8)	0.42
F	4 (28.6)	9 (29)	19 (42.2)	
Median age, y (IQR)	75.5 (59–83)	66 (49–74)	69 (60–76)	0.13
Ward of acquisition‡				
Outpatient	0	0	0	0.48
ICU/emergency	2 (14.3)	8 (25.8)	4 (8.9)	
Medicine	5 (35.7)	9 (29)	14 (31.1)	
Geriatrics	3 (21.4)	3 (9.7)	4 (8.9)	
Oncology	0	0	0	
Pediatrics/gynecology	0	0	0	
Surgery	4 (28.6)	8 (25.8)	18 (40)	
Other	0 (0)	3 (9.7)	5 (11.1)	
Microbiology				
CoNS	5 (35.7)	10 (32.3)	22 (48.9)	0.72
Staphylococcus aureus	3 (21.4)	6 (19.4)	7 (15.6)	
Enterobacterales	3 (21.4)	6 (19.4)	6 (13.3)	
Polymicrobial	1 (7.1)	5 (16.1)	8 (17.8)	
Other gram-negative	0 (0)	2 (6.5)	1 (2.2)	
Other gram-positive	1 (7.1)	1 (3.2)	0	
Fungi	1 (7.1)	1 (3.2)	1 (2.2)	
CRABSI attributed to short term CVC, n = 94§	N = 30	N = 37	N = 27	
Sex				
M	21 (70)	26 (70.3)	17 (63)	0.80
F	9 (30)	9 (29.7)	10 (37)	
Median age, y (IQR)	47 (0–65)	60 (50–67)	58 (53–65)	0.05
Ward of acquisition‡				
Outpatient	0 (0)	1 (2.7)	3 (11.1)	0.84
ICU/emergency	5 (16.7)	12 (32.4)	8 (29.6)	
Medicine	1 (3.3)	6 (16.2)	1 (3.7)	
Geriatrics	1 (3.3)	1 (2.7)	1 (3.7)	
Oncology	8 (26.7)	9 (24.3)	8 (29.6)	

Characteristics	2020	2021	2022	p value
Pediatrics/gynecology	12 (40)	2 (5.4)	4 (14.8)	
Surgery	2 (6.7)	5 (13.5)	2 (7.4)	
Other	1 (3.3)	1 (2.7)	0	
Microbiology				
CoNS	14 (46.7)	10 (27)	13 (48.1)	0.81
S. aureus	4 (13.3)	5 (13.5)	4 (14.8)	
Enterobacterales	4 (13.3)	4 (10.8)	2 (7.4)	
Polymicrobial	1 (3.3)	4 (10.8)	1 (3.7)	
Other Gram-negative	1 (3.3)	4 (10.8)	2 (7.4)	
Other Gram-positive	6 (20)	9 (24.3)	4 (14.8)	
Fungi	0 (0)	1 (2.7)	1 (3.7)	
CRABSI attributed to long term CVC, n = 74¶	N = 28	N = 23	N = 23	
Sex				
M	17 (60.7)	14 (60.9)	9 (39.1)	0.22
F	9 (29.3)	9 (29.1)	14 (60.8)	
Median age, y (IQR)	58.5 (46–70)	57 (31–65)	69 (45–77)	0.12
Ward of acquisition‡				
Outpatient	2 (7.1)	1 (4.3)	2 (8.7)	0.78
ICU/emergency	1 (3.6)	0 (0)	1 (4.3)	
Medicine	2 (7.1)	4 (17.4)	6 (26.1)	
Geriatrics	6 (21.4)	4 (17.4)	4 (17.4)	
Oncology	7 (25)	7 (30.4)	2 (8.7)	
Pediatrics/gynecology	3 (10.7)	3 (13)	1 (4.3)	
Surgery	5 (17.9)	2 (8.7)	4 (17.4)	
Other	2 (7.1)	2 (8.7)	3 (13)	
Microbiology				
CoNS	6 (21.4)	8 (34.8)	7 (30.4)	0.62
S. aureus	4 (14.3)	4 (17.4)	2 (8.7)	
Enterobacterales	6 (21.4)	4 (17.4)	6 (26.1)	
Polymicrobial	7 (25)	2 (8.7)	3 (13)	
Other gram-negative	0 (0)	2 (8.7)	0	
Other gram-positive	2 (7.1)	3 (13)	3 (13)	
Fungi	3 (10.7)	0 (0)	2 (8.7)	

*Values represent no. (%), except where indicated. BSI, bloodstream infection; CoNS, coagulase-negative staphylococci; CRABSI, catheter related/associated bloodstream infection; CRBSI, catheter-related BSI; CVC, central venous catheter; ICU, intensive care unit; PVC, peripheral venous catheter.
†Overall, we observed a total of 9 (10%) BSIs related to PVCs (CRBSI). Among CRABSI attributed to PVC, 14.3% (2/14) were related to PVCs during 2020, 3.2% (1/31) during 2021, and 13.3% (6/45) during 2022.
‡Wards of acquisition reported in this table belong to 1 of the 10 sites of Geneva University Hospitals, including 5 rehabilitation or palliative care sites, 1 acute care, 1 geriatric, 1 pediatric, 1 gynecology-obstetrics, and 1 psychiatry site.
§Overall, we observed a total of 57 (60.6%) BSIs related to short term CVCs (CRBSI). Among CRABSI attributed to short term CVC, 66.7% (20/30) were related to short term CVC during 2020, 54% (20/37) during 2021, and 63% (17/27) during 2022.
¶Overall, we observed a total of 54 (60.8%) BSIs related to long term CVCs (CRBSI). Among CRABSI attributed to long term CVC, 71.4% (20/28) were related to long term CVC during 2020, 47.8% (11/23) during 2021, and 60.9% (14/23) during 2022.

Appendix Table 2. Number of CRABSI, CRABSI attributed to PVC, short-term and long-term CVC, and patient-days, used for CRABSI incidence rate ratio calculations, Switzerland*

Year	Total no.†	No. attributed to PVC	No. attributed to short-term CVC	No. attributed to long-term CVC	Patient-days
2020	69	14	30	28	651,829
2021	85	31	37	23	645,666
2022	86	45	27	23	680,682

*BSI, bloodstream infection; CoNS, coagulase-negative staphylococci; CRABSI, catheter related/associated bloodstream infection; CVC, central venous catheter; PVC, peripheral venous catheter.
†The total number of CRABSI could be less than the sum of all CRABSI attributed to PVC, short-term CVC and long-term CVC. Nine cases were possibly attributable to >1 intravascular catheter.

Appendix Table 3. Number of catheters placed per year in a study of increased peripheral venous catheter bloodstream infections during the COVID-19 pandemic, Switzerland*

Characteristics	2020	2021	2022
Total no. inserted	108,491	111,363	86,615
Dwell time >96 h	19,839 (18.3)	19,660 (17.7)	14,690 (17.0)
Dwell time >120 h	10,195 (9.4)	9,315 (8.4)	6,543 (7.6)

*Values represent no. (%)

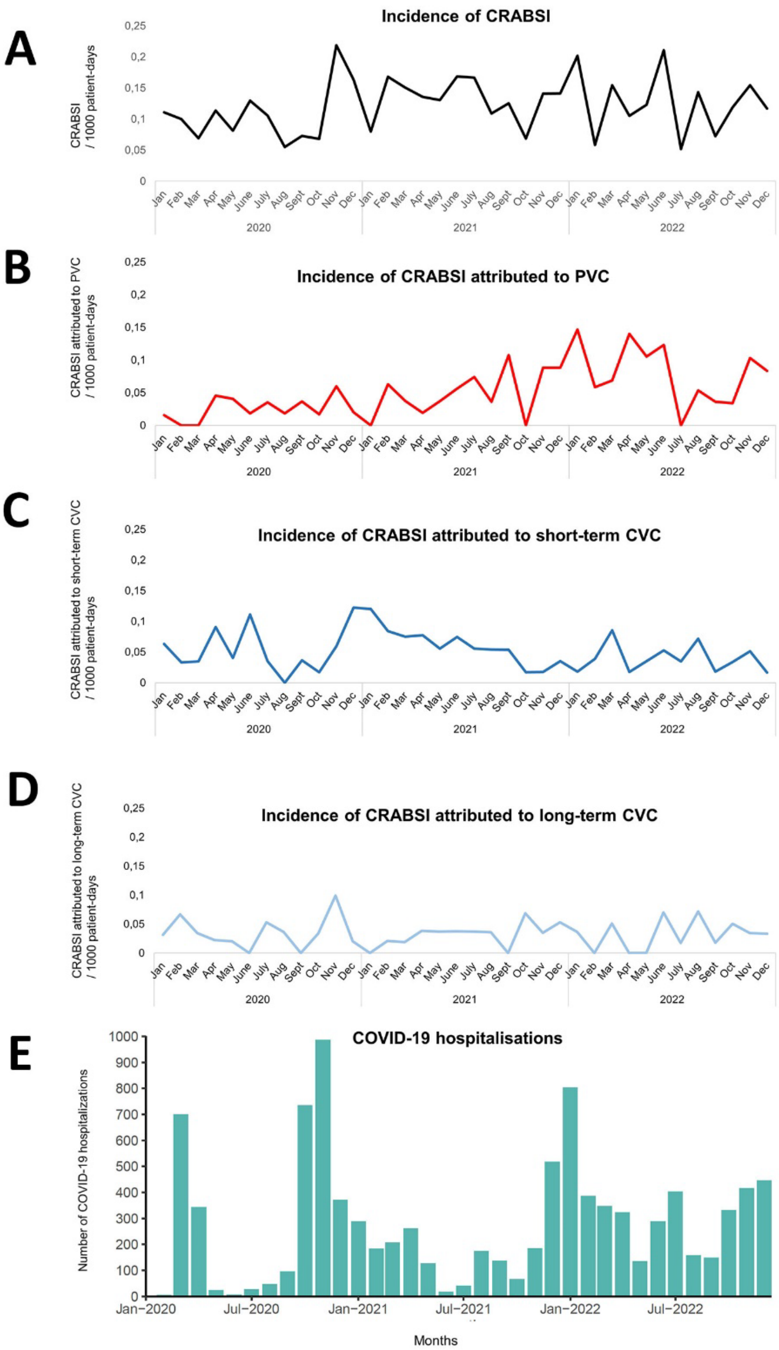

Appendix Figure 1. Monthly incidences of intravascular catheter bloodstream infections during the COVID-19 pandemic, Switzerland. A–D) Incidence of CRABSI per 1,000 patient-days; E) number of COVID-19 hospitalizations per month at Geneva University Hospitals, January 1, 2020–December 31, 2022. A) Overall CRABSI incidence; B) CRABSI incidence attributed to PVC; C) CRABSI incidence attributed to short-term CVC. D) CRABSI incidence attributed to long-term CVC. CRABSI, catheter related/associated bloodstream infection; CVC, central venous catheter; PVC, peripheral venous catheter.

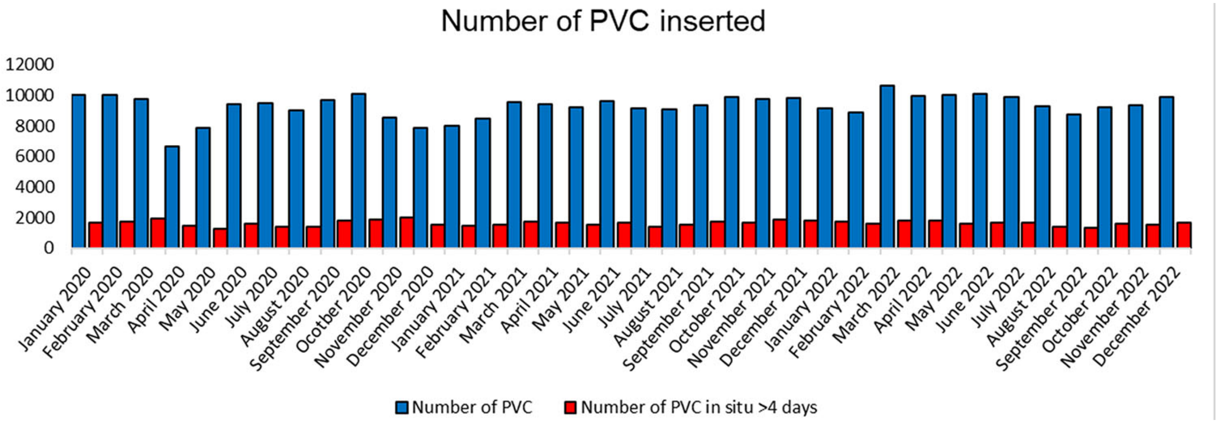

Appendix Figure 2. Total number of inserted PVC in a study of intravascular catheter bloodstream infections during the COVID-19 pandemic, Switzerland, January 1, 2020–December 31, 2022. Graph shows total PVC inserted and number in situ >96 hours. PVC, peripheral venous catheter.

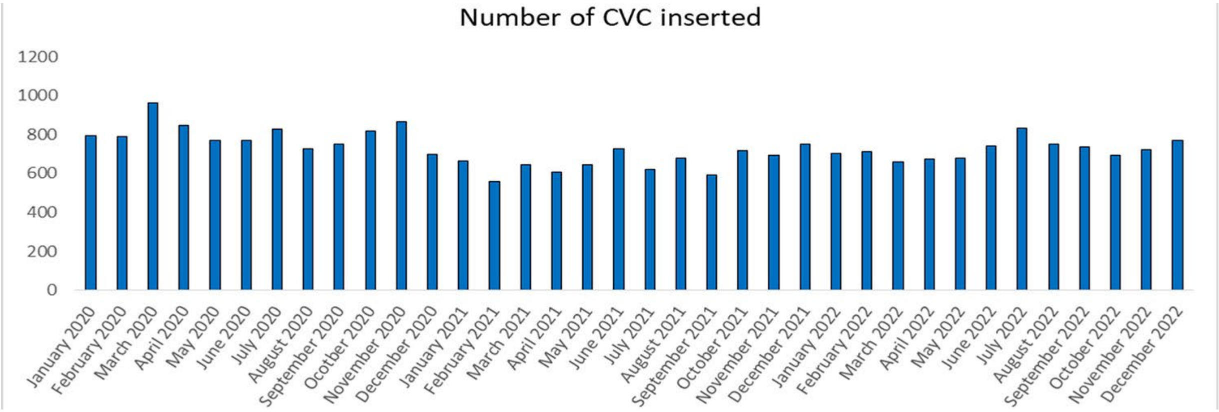

Appendix Figure 3. Total number of central venous catheters inserted per month in a study of intravascular catheter bloodstream infections during the COVID-19 pandemic, Switzerland, January 1, 2020–December 31, 2022. CVC, central venous catheter.

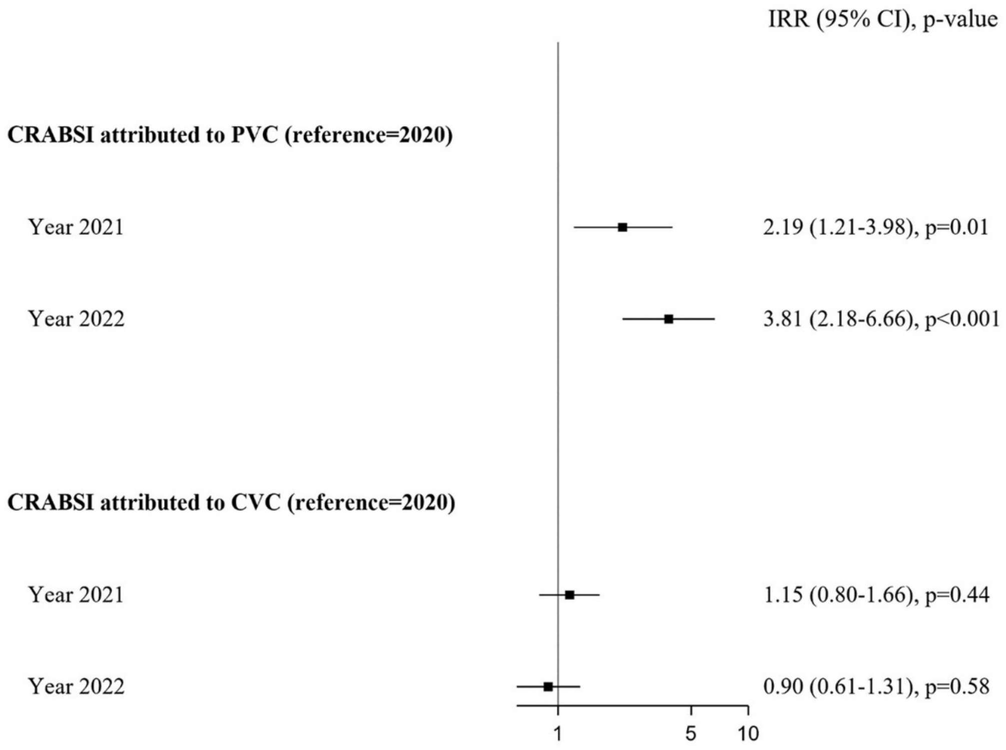

Appendix Figure 4. Incidence rate ratios for intravascular catheter infections, CRABSI attributed to PVC, and CRABSI attributed to CVC in a study of intravascular catheter bloodstream infections during the COVID-19 pandemic, Switzerland, January 1, 2020–December 31, 2022. Squares indicate IRR, bars indicate 95% CI. Short-term CVC and long-term CVC were considered together. Catheter-days were used as the denominator. CRABSI, catheter related/associated bloodstream infection; CVC, central venous catheter; IRR, incidence rate ratio; PVC, peripheral venous catheter.
